# Adrenalectomy for Cushing’s syndrome: do’s and don’ts

**Published:** 2016

**Authors:** DN Paduraru, A Nica, M Carsote, A Valea

**Affiliations:** * Carol Davila” University of Medicine and Pharmacy, Bucharest, Romania; Department of Surgery, University Emergency Hospital, Bucharest, Romania; ** ”Carol Davila” University of Medicine and Pharmacy, Bucharest, Romania; Department of Anesthesiology, University Emergency Hospital, Bucharest, Romania; *** ”Carol Davila” University of Medicine and Pharmacy, Bucharest, Romania; Department of Endocrinology, “C. I. Parhon” National Institute of Endocrinology, Bucharest, Romania; **** ”I. Hatieganu” University of Medicine and Pharmacy, Cluj-Napoca, Romania; Department of Endocrinology, Clinical County Hospital, Cluj-Napoca, Romania

**Keywords:** adrenalectomy, laparoscopy, Cushing’s syndrome, adrenal tumor

## Abstract

**Aim.** To present specific aspects of adrenalectomy for Cushing’s syndrome (CS) by introducing well established aspects (“do’s”) and less known aspects (“don’ts”).

**Material and Method.** This is a narrative review.

**Results.** The “do’s” for laparoscopic adrenalectomy (LA) are the following: it represents the “gold standard” for secretor and non-secretor adrenal tumors and the first line therapy for CS with an improvement of cardio-metabolic co-morbidities; the success rate depending on the adequate patients’ selection and the surgeon’s skills. The “don’ts” are large (>6-8 centimeters), locally invasive, malignant tumors requiring open adrenalectomy (OA). Robotic adrenalectomy is a new alternative for LA, with similar safety and conversion rate and lower pain drugs use. The “don’ts” are the following: lack of randomized controlled studies including oncologic outcome, different availability at surgical centers. Related to the sub-types of CS, the “do’s” are the following: adrenal adenomas which are cured by LA, while adrenocortical carcinoma (ACC) requires adrenalectomy as first line therapy and adjuvant mitotane therapy; synchronous bilateral adrenalectomy (SBA) is useful for Cushing’s disease (only cases refractory to pituitary targeted therapy), for ectopic Cushing’s syndrome (cases with unknown or inoperable primary site), and for bilateral cortisol producing adenomas. The less established aspects are the following: criteria of skilled surgeon to approach ACC; the timing of surgery in subclinical CS; the need for adrenal vein catheterization (which is not available in many centers) to avoid unnecessary SBA.

**Conclusion.** Adrenalectomy for CS is a dynamic domain; LA overstepped the former OA area. The future will improve the knowledge related to RA while the cutting edge is represented by a specific frame of intervention in SCS, children and pregnant women.

**Abbreviations:** ACC = adrenocortical carcinoma, ACTH = Adrenocorticotropic Hormone, CD = Cushing’s disease, CS = Cushing’s syndrome, ECS = Ectopic Cushing’s syndrome, LA = laparoscopic adrenalectomy, OA = open adrenalectomy, PA = partial adrenalectomy, RA = robotic adrenalectomy, SCS = subclinical Cushing’ syndrome

## Introduction

The field of adrenals surgery is complex, since the indication is established by an endocrinologist in addition to imagery and laboratory assessment, while the procedure itself is related to surgical procedures and adequate anesthesia. Techniques changed during decades and evidence based medicine provided a good support related to the types of endocrine tumors that needed adrenalectomy and associated outcome, highlighting Cushing’s syndrome (CS) as a key player in this domain[**1**,**2**].

## Aim

Our purpose is to present specific aspects of adrenalectomy for CS by introducing classical, well established aspects (“do’s”) as well as less known, accepted or used aspects (“don’ts”). 

## Material and Method 

This is a narrative review using a PubMed research. Several subtopics in the field of adrenalectomy were displayed related to either surgical or endocrine issues. 

## Results

***Laparoscopic adrenalectomy (LA)***

LA represents the current choice since it is a safe, effective, minimally invasive, well tolerated procedure with a short hospital stay due to early recovery and control of tumor-related hormonal excess, being generally regarded as “gold standard” for secretor and non-secretor adrenal tumors [**3**-**5**].Adrenalectomy is the first line therapy for CS with an improvement of classical cardio-metabolic co-morbidities as high blood pressure, diabetes mellitus, obesity, which is registered early after tumor removal and continues in time[**6**-**8**]. LA approach is either intraperitoneal or retroperitoneal; sub-mesocolic access has a shorter time and a less extended dissection; the key to a successful procedure is an adequate patients’ selection and surgeon’s skills and experience regarding the anatomy’s knowledge, the choice of approach, delicate tumor handling and efficient hemostasis, as well as optimal anesthesia and monitoring of vital parameters including immediately after surgery, due to prior complications of hypercortisolemia[**9**-**11**].The controversies about LA are related to tumors larger than 5-6 centimeters, which are not safely removed, according to some studies, due to a potential malignant behavior [**12**-**14**].LA performed in extended tumors takes more time, it associates a higher conversion rate, but many authors agree that in selected cases it is feasible by an experienced surgeon[**15**-**17**].The peri-adrenal invasion as found in kidney, the local lymph nodes spreading and a tumor diameter higher than 8 centimeters mostly recommend avoiding LA[**18**-**20**].

***Open adrenalectomy (OA***)

The indications of OA are represented by the limits of LA but this modern frame is different to what was considered two decades ago since LA first description in 1992 [**21**-**23**].The well-known disadvantages of OA are higher blood loss and transfusion rate, local postoperative pain, longer hospitalization, delayed resumption of oral intake, esthetical considerations[**24**-**26**].When comparing OA to LA, the only advantages are a lower time of surgery and visual control of the anatomical area, which is imperiously needed when oncologic aspects are involved, especially in large adrenal masses or cysts (cystic tumors or mix cyst - solid tumors) but, even in these particular cases, the balance between doing and not doing OA depends on a complex, adequate pre-operatory evaluation and a skilled surgeon[**27**-**30**].

***Robotic adrenalectomy (RA)***

RA is a new particular type of procedure, mainly regarded as an alternative to LA, which still needs safety profile data on large population studies, but currently, different etiologies such as aldosteronoma, pheochromocytoma, adrenal CS, are approachable[**31**-**33**].RA allows less pain medication, it associates similar safety, and conversion rate as LA and potentially (but not all the authors agree) equal costs to LA[**34**-**36**].Thefirst case of bilateral RA for CS was published in 2014, while OA and LA for similar conditions are already published on large series [**37**,**38**].The limits of RA are the following: lack of substantial statistical data (randomized controlled studies); the availability in different surgical centers all over the world and the big “don’t” is the lack of evidence regarding the oncologic outcome of malignant adrenal tumors[**39**,**40**].

***Partial adrenalectomy (PA)***

PA is preferred for children and in cases of bilateral conditions (mostly gene-related) because the removal of both adrenals will cause adrenal insufficiency [**41**-**43**].However, nowadays, a limited use of PA for CS has been seen in macronodular bilateral hyperplasia or synchronous cortisol producing adenomas [**44**-**46**].

***Adrenal Cushing’s syndrome***

CS caused by a cortisol secreting adrenal tumor associates a higher risk of arterial hypertension, weight gain, impaired glucose profile, dyslipidemia, ischemic heart disease, stroke, osteoporosis, myopathy, hypogonadism, which are remitted after the resection, depending on the patient’ age, prior conditions, time of hypercortisolemia exposure, etc. [**47**-**49**].LA is preferred as technique; the cure of condition is expected and, in order to have a good outcome, a collaboration between the endocrinologist, cardiologist, surgeon and anesthetist is needed to select the patient, control the cardio-metabolic anomalies before, during and after surgery and to minimize the impact of surgery[**50**-**52**].Opposite to Cushing’s disease, when medical therapy is eventually needed in many cases, pre-operative medication to lower cortisol levels,such as ketoconazole or metyrapone, is not needed since the patient is directly referred to surgery[**53**-**55**].

***Subclinical Cushing’s syndrome (SCS)***

SCS represents a modern concept of persistent mild hypercortisolemia usually related to a cortisol producing adenoma (up to one third of adrenal incidentalomas) but controversies are related to definition (for instance, the cut offs of cortisol after dexamethasone suppression test) and management (whether to perform or not adrenalectomy) [**56**-**58**].Successful adrenal removal is usually followed by transitory adrenal insufficiency on 50% of the cases depending on prior hypercortisolemia severity[**59**,**60**].From the point of view of the approach, LA is preferred (morbidity rate of 0.8%); surgery controls cortisol excess and brings a partial improvement of cardio-metabolic complications but the results are heterogeneous, that being the reason why not all the clinicians recommend surgery[**61**-**63**].

***Adrenocortical carcinoma (ACC)***

ACC is a rare (an incidence of 0.5-1 cases/million/year), severe malignancy; adrenalectomy is the first line therapy and it has the potential of curing the condition that is otherwise not done by the adrenolytic agent mitotane or chemotherapy[**64**-**66**]. ACC may develop CS, which is resumed after tumor is operated in some cases [**67**,**68**]. OA is preferred; a careful pre-operative staging improves the prognosis while “don’ts” aspects are related to choosing the right surgeon and the surgeon’s choices since in this particular type of cancer the prognosis of the approach, the extent of surgery, and the disease-free resection margins, are essential[**69**-**71**].

***Cushing’s disease (CD)***

CS includes the cases of CS caused by an ACTH (Adrenocorticotropic Hormone) producing pituitary adenoma to which most of the therapeutical options are targeted, such as selective hypophysectomy, pituitary irradiation (especially gamma knife), new drugs, such as dopamine agonist cabergoline and modern somatostatin analogue pasireotide[**72**-**74**].Bilateral adrenalectomy is rarely used these days and it has become optional for refractory CS to traditional and modern management when control of hypercortisolism state is imperiously needed because of associated high rate of morbidity and mortality[**75**-**77**].Currently, bilateral LA is preferred to OA; the hormonal excess is turned into a hormonal deficiency meaning that post-operatory chronic adrenal insufficiency is a life threatening condition, which requires glucocorticoid and mineralocorticoid lifelong substitution therapy and, also, a specific, rare complication, such as Nelson’s syndrome, may appear [**78**-**80**]. Nevertheless, adrenalectomy for CD subscribes for “do’s” aspects unless all the other numerous surgical and pharmacological tools are inefficient, contraindicated, or not available [**81**,**82**].

***Ectopic Cushing’s syndrome (ECS)***

ECS, covering 10 to 20% of all CS cases, is an epiphenomenon in cancers like lung and pancreatic carcinoma, neuroendocrine tumors, medullar thyroid cancer, requiring a large panel of investigation tools for an adequate recognition of the causing tumor[**83**,**84**].Bilateral adrenalectomy is recommended only for the cases without the identification of the primary cause or without operable cancers in which steroidogenesis inhibitors, such as ketoconazole, metyrapone, etomidate, mitotane, are not efficient in controlling the hypercorticism and its metabolic complications; thus, adrenal surgery become a life saving procedure under these specific circumstances despite the associated high anesthetically risks [**85**-**87**].

***Bilateral adrenal masses***

A part from the macronodular transformation of both adrenals due to ACTH stimulation, as seen in CD and ECS, CS-related bilateral masses are found in genetic syndromes (such as pigmented macronodular hyperplasia or Multiple Endocrine Neoplasia type syndrome 1), double cortisol producing adenoma or unilateral hormonally active tumor with a contra-lateral incidentaloma[**88**-**90**].Synchronous LA is safely done; cortex-sparing surgery may be tried; RA represents an alternative to LA [**91**,**92**].The “don’ts” aspects include avoiding the removing of both glands in unnecessary situations, knowing the life impact of adrenal insufficiency and this may be prevented by performing ample pre-operative investigations, including, if necessary and available, adrenal vein catheterization[**93**,**94**]. In cases with bilateral adrenal tumors-related CS without an adequate identification of cause, unilateral adrenalectomy of the largest mass followed by re-evaluation is advisable [**95**,**96**].

***Adrenalectomy associated with surgical cure for non-endocrine conditions***

If the right adrenal is removed, synchronous cholecystectomy is feasible for indications such as gallbladder stones[**97**]. Large oncologic interventions allow the dissection of pancreas and adrenal glands [**98**]. Nephrectomy for renal cancer may require the adrenal removal if glandular spreading is found (with a prevalence of less than 0.6% in early stages and around 8% in advanced renal cancer)[**99**].

***Adrenalectomy in pediatric population***

CS of any mentioned types may be exceptionally found in children and adolescents and adrenalectomy represents a challenge[**100**].Except forOA, which is necessary in ACC and large tumors, minimally invasive adrenalectomy is the choice (retroperitoneoscopicapproach is promising but the most common is transperitoneal lateral laparoscopy); the preserving of the adrenal cortex might help; RA is controversial[**101**,**102**].

***Adrenalectomy during pregnancy***

The diagnosis of CS is uncommon during pregnancy (the most frequent cause is an adrenal adenoma) associating an increased maternal and fetal risk of complications and death[**103**,**104**]. If an adequate medication does not allow postponing the surgery after birth, laparoscopic adrenalectomy may be performed during the third trimester; an experienced surgeon as well as a multidisciplinary team including anesthetist, obstetrician and neonatology specialist,is needed [**105**,**106**].

## Discussion

Adrenal insufficiency may appearafter adrenalectomy for post-operatory CS, representing a potential life-threatening situation if adequate intravenous corticoids are not provided during and immediately after adrenalectomy followed by oral supplements as soon as their ingestion is possible[**107**,**108**]. The condition requires lifelong substitution unless contra-lateral gland recovers, which is seen within 6 months up to 2 years (maximum 4-5 years); time to recovery mostly depends on CS etiology, it is shorter in younger patients and it seems independent of sex, pre-operative symptoms and levels of cortisol or ACTH; after adrenal surgery of one side, CS may persist in cases of ECS or CD[**109**,**110**].

## Conclusion

The adrenalectomy for CS is a dynamic domain; LA overstepped the former OA area, which is still needed in extremely large aggressive tumors; synchronous adrenalectomy is feasible for CS, ECS and bilateral adrenal cortisol secreting adenomas while special populations, such as pregnant women and children, may also be candidates for this type of surgery. In future, the knowledge related to RA will improve, while the cutting edge is represented by a specific frame of intervention in SCS, pediatric population, etc

#The authors have an equal contribution to the article.

**Acknowledgement**

None.

**Conflict of interest**

The authors have nothing to declare.
